# Human‐Guided Bayesian Optimization Enables High‐Throughput Laser Annealing of Mesoporous SiO_x_ Anodes for Lithium‐Ion Batteries

**DOI:** 10.1002/advs.76607

**Published:** 2026-07-17

**Authors:** Chaeyoung Park, Yeongje Lee, Sunho Jeong, Eungkyu Lee

**Affiliations:** ^1^ Department of Electronic Engineering Kyung Hee University Yongin‐si Gyeonggi‐do Republic of Korea; ^2^ Department of Materials Science and Engineering Kyung Hee University Yongin‐si Gyeonggi‐do Republic of Korea

**Keywords:** active learning, bayesian optimization, empirical approach, laser annealing, lithium‐ion batteries, silicon suboxide anode

## Abstract

Silicon suboxide (SiO_x_) has attracted significant attention as a promising anode material for next‐generation lithium‐ion batteries due to its high capacity and improved structural stability. However, precise control over stoichiometry and pore structure remains challenging under conventional thermal annealing. Herein, we introduce an empirical‐aided active learning (EAAL) approach to optimize high‐throughput laser‐induced photothermal annealing of high‐performance SiO_x_ anodes. By synergistically combining probabilistic machine learning with empirical domain knowledge, the EAAL framework efficiently explores complex processing parameter spaces using a limited number of experiments. Remarkably, the EAAL‐optimized anode achieves electrochemical performance comparable to that of an empirically optimized counterpart while significantly reducing the number of laser irradiation passes from five to two, thereby improving practical production throughput in cumulative laser‐processing. Furthermore, the optimal conditions are identified using only 26 experimental data points, significantly improving process efficiency. This study demonstrates a data‐efficient strategy for process optimization by synergistically integrating machine learning with empirical domain knowledge and provides a scalable pathway for the advanced manufacturing of high‐performance energy storage materials.

## Graphical abstract

An empirical‐aided active learning framework is developed to optimize high‐throughput laser‐induced photothermal annealing of silicon suboxide anodes. By integrating probabilistic machine learning with empirical domain knowledge, this approach achieves optimal electrochemical performance using limited experiments. It effectively reduces the required laser passes from five to two, thereby improving production throughput.

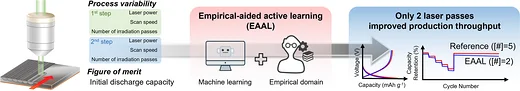

## 1 Introduction

The increasing demand for portable electronic devices and electric vehicles has accelerated the development of energy storage systems with higher energy density and long cycle life. Lithium‐ion batteries (LIBs) have emerged as the dominant energy storage technology due to their high energy density and reliable cycling performance. However, the electrochemical performance of LIBs is approaching the practical limit of conventional graphite anodes (372 mAh g^−1^) [1, 2, 3, 4, 5, 6, 7]. Silicon has been widely regarded as one of the most promising alternative anode materials due to its exceptionally high theoretical capacity (3579 mAh g^−1^) [8, 9, 10]. However, its practical application is severely limited by large volume expansion during lithiation and delithiation, resulting in particle pulverization and rapid capacity fading [11, 12]. To address these challenges, silicon suboxide (SiO_x_) has attracted significant attention as an alternative anode material, as the presence of the oxide matrix can buffer volume changes and enhance structural stability during repeated cycling [13, 14].

The electrochemical performance of SiO_x_ anodes strongly depends on their stoichiometry (O/Si) and internal pore structure [15, 16, 17, 18, 19, 20]. A reduction reaction at elevated temperatures generates electrochemically active low‐valence Si species that serve as reversible lithiation sites, while the formation of internal porous structures helps accommodate volume expansion during cycling. Among various approaches, carbothermal reduction using carbon as a reducing agent has been investigated because it can simultaneously induce chemical reduction and internal pore formation through gas evolution (e.g., CO/CO_2_) [21, 22]. However, conventional thermal annealing typically requires an extremely high thermal budget, such as temperatures above 800°C for a prolonged time of over 12 h, which inevitably results in unwanted surface re‐oxidation reactions, limiting controllability over the stoichiometry [23, 24].

In contrast, laser‐induced photothermal annealing can enable rapid and localized thermal processing. By exposing a confined region to strong optical energy, laser irradiation can induce efficient thermochemical reactions with well‐defined spatial and temporal control and ultrafast heating/cooling cycles [25]. As a result, laser‐based techniques have been widely applied in the research fields of materials synthesis and device fabrication. For example, laser‐induced photothermal processing has been reported to promote conductive network formation and oxide reduction through localized thermochemical reactions [26, 27, 28, 29, 30, 31, 32]. Advantageously, laser annealing also allows multiple irradiation sequences to control each thermochemical reaction within individual irradiation steps.

In our previous study [33], this stepwise photothermal evolution was realized in SiO_x_ anodes (with a specific capacity of 2139 mAh g^−1^ and an areal capacity of 9.5 mAh cm^−2^ at a mass loading of 6.6 mg cm^−2^) through two‐step irradiation sequences. The first sequence established a thermally and electrically conductive carbonized binder framework, while the second sequence promoted uniform carbothermal reduction and mesopore formation within the SiO_x_ active material throughout the electrode. In general, the number of irradiation passes in each sequence inevitably increases to maximize the desired annealing effects, leading to longer cumulative processing times that are unfavorable for production efficiency in practical applications. Despite substantial efforts to reduce the number of irradiation passes and enhance process throughput without compromising performance, empirical trial‐and‐error (ETAE) approaches alone cannot efficiently identify reduced‐pass conditions [34, 35, 36]. The electrochemical response of laser‐annealed SiO_x_ anodes is governed by complex irradiation parameters, including laser scan speed, laser power, and the number of irradiation passes. These coupled parameters define the local thermal history and thus determine the extent of SiO_x_ reduction, pore evolution, and conductive network formation within the electrode. As the dimensionality of this processing space increases, exhaustive empirical exploration becomes impractical. Consequently, optimization of laser annealing conditions remains challenging using labor‐intensive ETAE approaches.

To overcome the limitations of ETAE methods, researchers have increasingly adopted probabilistic machine learning strategies, such as Gaussian process regression (GPR). These approaches have been successfully applied across various electronic device fields, ranging from thin‐film transistors to thermoelectric devices [37, 38]. The primary goal of these techniques is to establish a quantitative relationship between input processing parameters and measured outputs—typically defined as a figure of merit (FoM)—using a limited amount of experimental data. Once this relationship is modeled, the algorithm can efficiently predict the optimal parameter values required to maximize the FoM. For instance, Javash et al. [37] used Bayesian optimization (BO) to identify optimal laser‐annealing conditions for silver‐selenide thermoelectric films in fewer than forty experiments. Similarly, Lee et al. [38] employed BO to optimize deposition conditions for dual‐layer oxide thin‐film transistors using only 15 experiments. Although probabilistic machine learning can reduce experimental costs, significant limitations remain. Previous studies typically relied on a single kernel function (e.g., the radial basis function, RBF) alongside fixed hyperparameter optimization methods like maximum likelihood estimation (MLE). This restrictive approach limits the flexibility and predictive accuracy of the surrogate models used in BO. Furthermore, conventional BO frameworks generally fail to incorporate prior empirical knowledge, which continues to play a crucial role in guiding advancements in materials science.

Herein, we introduce an empirical‐aided active learning (EAAL) approach to optimize laser irradiation parameters for high‐performance SiO_x_ anodes (Figure 1). Unlike conventional BO or purely empirical optimization, which solely rely on either machine learning algorithms or ETAE, the EAAL framework synergistically combines the strengths of both approaches. During each optimization iteration, four distinct GPR models are constructed using different kernel functions (e.g., RBF, Matern) and hyperparameter optimization techniques (i.e., maximum likelihood estimation and maximum a posteriori (MAP)) [39]. Each model then proposes potential candidates for the optimal fabrication conditions. Subsequently, empirical domain knowledge is applied to select the most promising candidates for experimental verification. Remarkably, the EAAL‐optimized anode requires only two laser irradiation passes, yet achieves an electrochemical performance comparable to that of an ETAE‐optimized anode that requires a highly complex five‐pass irradiation process. Consequently, the EAAL framework successfully discovers the optimal conditions using only 26 experimental data points. Compared to the ETAE‐optimized anode, the EAAL‐optimized anode retains 91.9% of the initial discharge capacity, 96.3% of the initial Coulombic efficiency, and 100% of the long‐term cycling stability. Furthermore, we investigated which specific GPR model best captures the complex parametric space of the SiO_x_ anode, and experimentally verified these quantitatively defined parameter landscapes.

**FIGURE 1:**
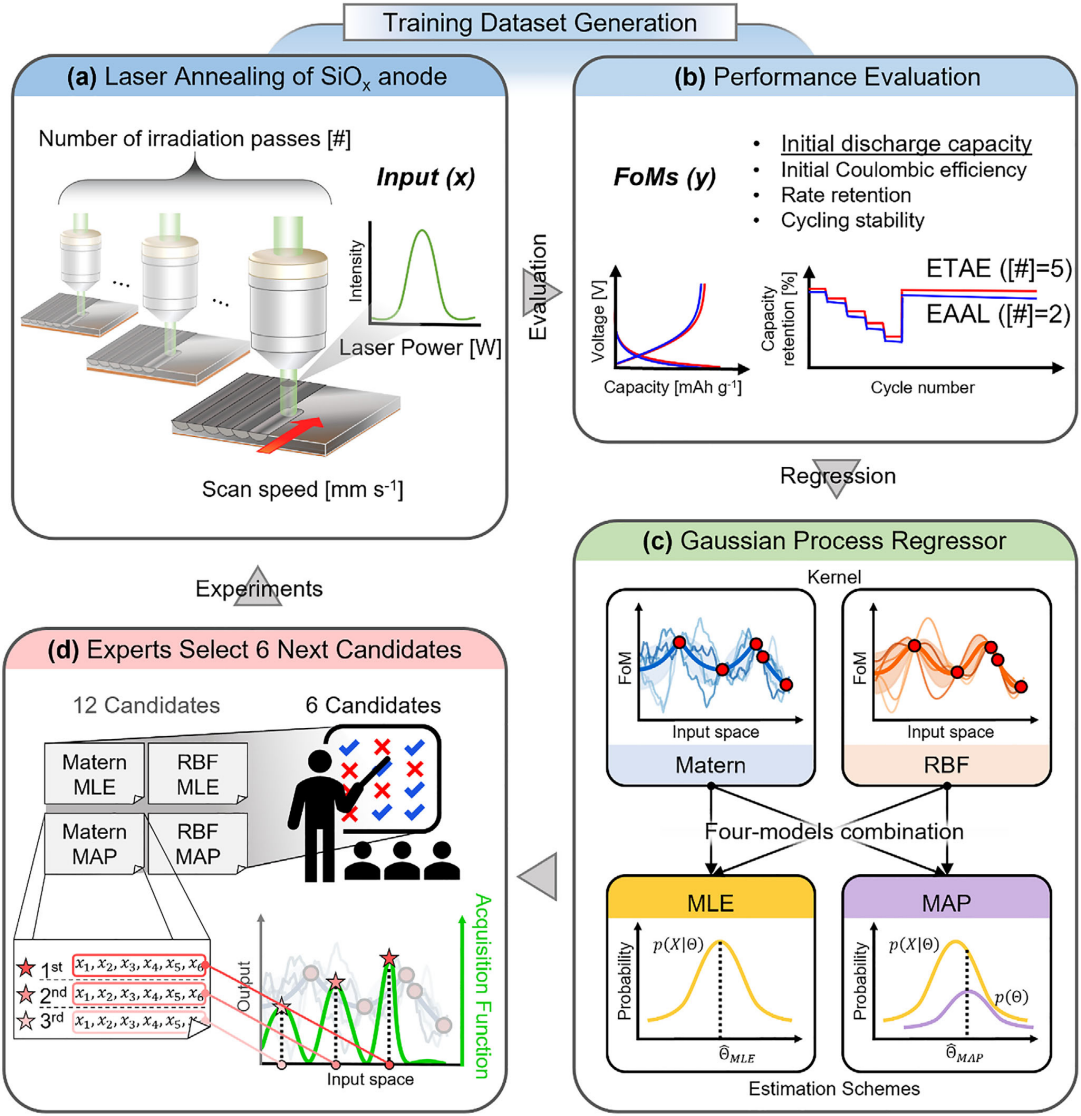
Schematic illustration of the EAAL framework | (a) The SiO_x_ anodes are fabricated with input variables (laser power, scan speed, and number of irradiation passes) (b) The electrochemical performance of the fabricated SiO_x_ anode is evaluated by half‐cell tests, yielding an output value (i.e., FoM). (c) A training dataset consisting of input variables and FoMs is constructed. Using this training dataset, four GPR models are trained with two kernels and two estimation schemes to predict the initial discharge capacity as the FoM over the input variables. (d) Acquisition functions (e.g., expected improvement) are evaluated for each model to generate candidate points (three per model, 12 in total), which are subsequently reduced to six through expert selection. (a–d) constitute one iteration and are repeated. See the main text for details.

## 2 Results and Discussion

### 2.1 EAAL Framework and Machine Learning Concept

The one optimization iteration of EAAL consists of four steps as follows. First, initial variable sets are empirically selected, where each set has six variables (e.g., 1st laser power, 1st scan speed, number of 1st irradiation passes, 2nd laser power, 2nd scan speed, and number of 2nd irradiation passes), corresponding to the SiO_x_ reduction process by laser annealing illustrated in Figure 1a. The laser power was varied from 1.8 to 4.5 W, while the scan speed ranged from 12.7 to 1 270 mm s^−1^. Samples were fabricated under these conditions, and their performance was characterized in terms of the initial discharge capacity, as illustrated in Figure 1b. The initial discharge capacity reflects the degree of carbothermal reduction and electrochemically active low‐valence Si formation in the SiO_x_ anode. Therefore, it was selected as the optimization objective in the EAAL framework. The capacity was normalized by 2139 mAh g^−1^ and expressed as a percentage. It is noted that the laser power and scan speed are normalized to percentile units in the EAAL process (e.g., the laser power is normalized by 30 W, and the scan speed by 1270 mm s^−1^). These variable sets and associated performance parameters form input–output pairs, constructing the training dataset. It is noted that the number of input variable sets is 2.5 ( 10^7^. Second, GPR models are used to recommend candidate optimal variable sets (e.g., twelve candidates), where the model constructs a surrogate of the parameter space by quantitatively learning the relationship between input and output values from the training dataset (Figure 1c). Third, an empirical approach selects six candidates from the twelve candidates, as shown in Figure 1d. Fourth, SiO_x_ anodes are fabricated with the selected variable sets, and the associated output performances are characterized experimentally. For the next optimization iteration, these experimental data points are added to the training dataset, and the second, third, and fourth steps are repeated until a stopping criterion (e.g., a targeted performance value) is satisfied.

A GPR model consists of a mean function and a covariance function—also known as a kernel. The kernel determines how the GPR model quantitatively describes unexplored regions of the parameter space (i.e., the relationship between the input variables and output performances) based on experimentally observed data [40]. The optimal candidates suggested by BO depend on the type of kernel used and its hyperparameters. Thus, using a single GPR model may not appropriately account for the unexplored parameter space. To mitigate this issue, we used four GPR models constructed from combinations of two kernel types (RBF and Matern) and two approaches for training the kernel hyperparameters (MLE and MAP), as depicted in Figure 1c. The acquisition function, which extracts the next candidate values for BO, quantifies the candidates that are capable of maximizing the initial discharge capacity across the four models. Among acquisition functions, expected improvement (EI) is used, which quantifies the potential to find a value superior to the maximum value observed thus far [41]. For each GPR model, the top three candidates with the highest EI values are extracted.

The RBF and Matern kernels encode different prior assumptions about the smoothness of the surrogate model. Thus, even with identical process conditions and experimental results, their regression outputs can differ. The RBF kernel assumes infinitely smooth functions across the process parameter space, producing nearly convex changes between training data points. This implies that capacity predictions remain stable even for moderate shifts in laser parameters (e.g., laser power from 1.8 to 2.4 W with other variables fixed), effectively assuming gradual material property transitions typical of diffusion‐dominated processes. In contrast, the Matern kernel accommodates rougher function forms—better suited for abrupt phase changes or defect formation in thin‐film deposition—and can therefore exhibit more abrupt changes in response to input variations compared to the RBF kernel. The detailed procedure for hyperparameter tuning using MLE or MAP is described in the Methods section.

We used MLE or MAP for training the hyperparameters of each kernel. Given a training dataset D and a set of model hyperparameters θ, the likelihood is defined as p(D│θ). In the MLE approach, the hyperparameters are optimized such that the value of this likelihood is maximized. In the MAP approach, the hyperparameters are selected to maximize the posterior distribution p(θ│D) by multiplying this likelihood by the prior distribution of the model parameters. A log‐normal distribution is used as the prior for the kernel hyperparameters (i.e., the hyperparameters are modeled as random variables that follow a log‐normal distribution). The key difference between these two approaches is that MLE relies solely on the information in the experimental data, whereas MAP additionally incorporates prior knowledge to regularize the hyperparameters. Thus, MLE can avoid potential bias arising from prior misspecification while MAP can lead to more stable estimates of both the mean prediction and its deviation (the predictive uncertainty of experimental output values).

### 2.2 GPR Regression and EAAL Optimization Process

The EAAL used five starting variable sets, which were systematically selected from 30 randomly generated candidates based on domain expertise to ensure physical feasibility and maximize diversity (see the Methods section for detailed selection criteria). At each optimization iteration, four probabilistic machine learning models predict twelve candidates (i.e., three candidates per model), from which six are selected for experimental verification. Specifically, for each model the candidate with the highest EI value is mandatorily selected, yielding four acquisition‐dictated candidates; from the remaining eight candidates, domain experts then select two based on the physical feasibility criteria described in Methods Section 4.2, giving a 4 + 2 = 6 selection per iteration. For the 4th iteration, as the optimization approached saturation, an adaptive probability of improvement (PI)—an acquisition function that prioritizes exploitation—was additionally considered alongside EI to aid the selection. The complete evaluation log for all 12 model‐suggested candidates at each iteration—including the predictive mean, uncertainty, acquisition values, and the physical justifications for their acceptance or rejection—is provided in Table S1. The EAAL process targets an initial discharge capacity exceeding 95%. However, rather than terminating immediately upon discovering a single high‐performing sample, the iterations continue until the acquisition functions indicate that the model uncertainty has sufficiently converged, ensuring both robust optimization and the statistical reliability of the response surface. In Figure 2a, the EAAL optimization results are summarized, where the predicted and measured initial discharge capacities of selected candidates per iteration are plotted as a function of each sample number (i.e., dataset number). The detailed optimization history, including the model predictions and EI values for these evaluated candidates, is summarized in Table S2. Figure 2b shows the values of process variables for each candidate. It is worth noting that while excluding failed samples ensured high accuracy of the response surface near the optimal region, it inherently limited the model's capability for generalized, full‐space exploration, since the surrogate did not directly learn the infeasible boundary; this feasibility information was instead supplied within each iteration through the expert filter. Future studies could address this limitation by employing constrained BO with a learned feasibility model to autonomously learn global feasibility boundaries without contaminating the regression surface.

**FIGURE 2:**
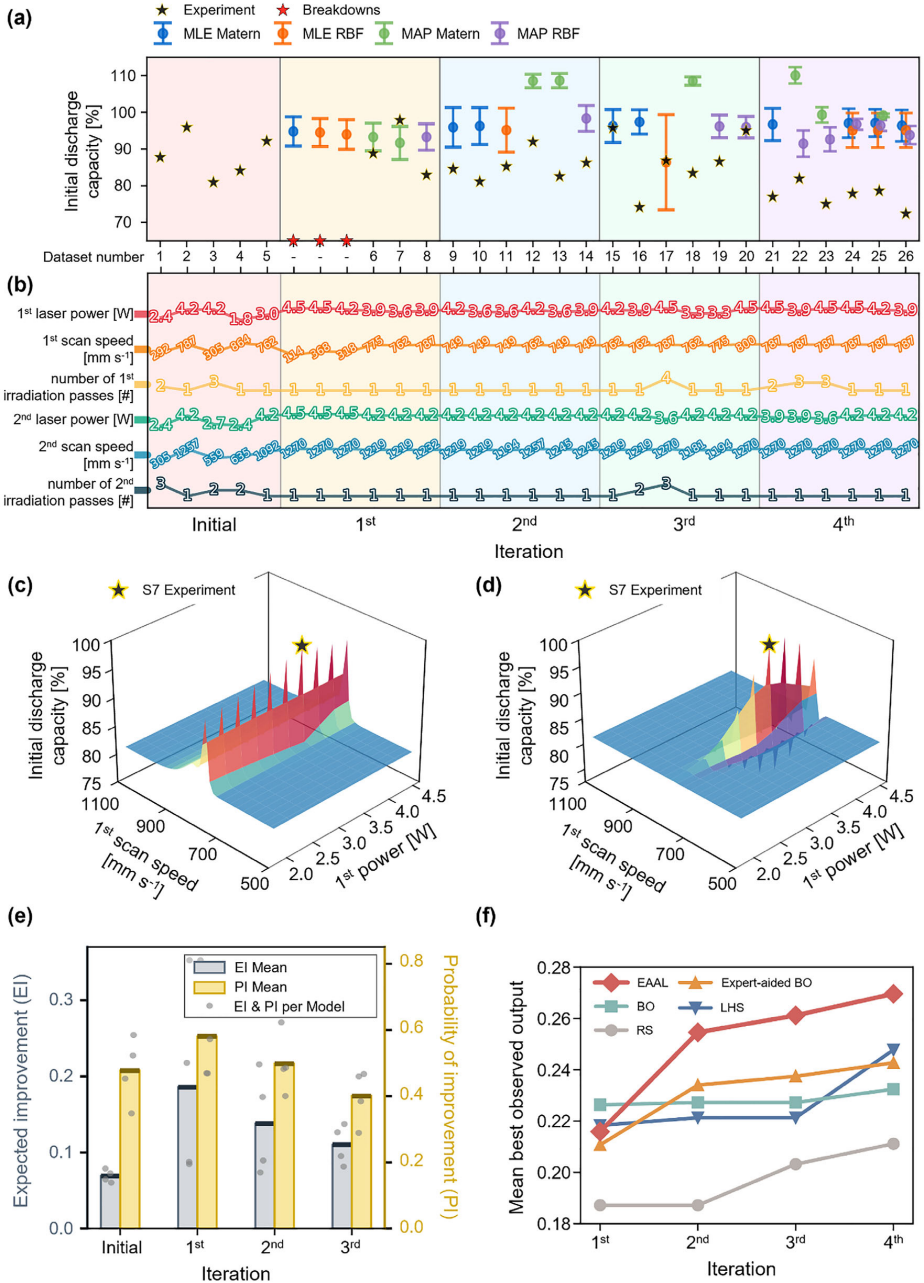
Optimization SiO_x_ anode using the EAAL framework | (a) Predicted and measured initial discharge capacity of selected candidates at each optimization iteration. The initial discharge capacity is normalized to 2139 mAh g^−^^1^ and expressed in percentile units. The horizontal axis indicates the index of samples in order of the optimization iterations. (b) Input variable values for the corresponding samples in (a). (c,d) Predicted initial discharge capacity as a function of 1st scan speed and 1st power calculated using (c) MLE Matern and (d) MLE RBF models after the 4th iteration. (e) EI and PI values from the four GPR models as a function of optimization cycle. Note that the 4th optimization cycle is the final one; therefore, EI and PI were not evaluated for this cycle. The mean EI and PI are shown as the gray and yellow bars, respectively. (f) Trajectory comparison under a shared oracle and identical experimental budget. The best initial discharge capacity is shown for five optimization strategies over four rounds of batch selection. Each curve represents the mean best initial discharge capacity averaged over five independent runs.

In the first iteration, the MAP Matern and MAP RBF models suggested three candidates with 1st scan speeds (762–787 mm s^−1^) and 1st laser powers (3.6–3.9 W). These three samples (S6, S7, and S8) were successfully fabricated, exhibiting initial discharge capacities of 97% for S7, 90% for S6, and 82% for S8. The measured values of samples S6 and S7 agree well with the predicted ranges from the MAP Matern model. We attribute this favorable outcome primarily to the initial candidates S2 and S5. In contrast, three other candidates suggested by the MLE Matern and MLE RBF models were non‐functional during characterization (red star symbols in Figure 2a). This may arise from the combination of relatively slower 1st scan speed (114–368 mm s^−1^) with higher 1st laser power (4.2–4.5 W), which can lead to sample breakdown due to overdosing in the photothermal process. It should be noted that three non‐functional samples generated in the initial stages, which suffered physical damage due to excessive photothermal energy, were excluded from the GPR training dataset. Assigning arbitrary zero or penalty values to these failed observations would act as severe outliers, distorting the spatial correlation of the surrogate model and degrading its predictive accuracy within the functional parameter space [4]. To prevent the acquisition function from continuously exploring these physically invalid regions—a known limitation of conventional unconstrained Bayesian optimization—the expert filtering step actively served as a physical constraint boundary, excluding candidate points that fell into regimes prone to thermal failure. While implementing a mathematically formalized constrained BO can present a promising avenue for fully automated systems in future studies, the human‐guided expert filter effectively managed this feasibility boundary within the current study.

At the second optimization iteration, the four GPR models suggested candidates with similar input variables: 1st laser powers of 3.6–4.2 W, 1st scan speeds of 749–762 mm s^−1^, 2nd laser power of 4.2 W, 2nd scan speeds of 1194–1245 mm s^−1,^ and a single irradiation pass for each scan. The predictions of these input variables can be understood by considering the inclusion of samples S6, S7, and S8. In the third iteration, the EAAL identified improved candidates with higher prediction accuracy compared to the previous iteration. The fabricated samples S15 and S20 exhibited measured initial discharge capacities above 95%, which lie within the predicted ranges from the MLE Matern and MAP RBF models. Sample S17, predicted by the MLE RBF model to have an initial discharge capacity of 87%, also agreed well with the measured value. On the other hand, sample S16 from the MLE Matern model, which used two irradiation passes in 2nd scan, showed an initial discharge capacity of only 75%, markedly lower than S15 and outside the predicted range, highlighting the critical influence of the number of irradiation passes in 2nd scan.

In the fourth optimization iteration, which we intend to be the last iteration, the EAAL suggested candidates with noticeable variation in the number of irradiation passes in the 1st scan. The 1st laser power (or 2nd laser power) values were 3.9–4.5 W (or 3.6–4.2 W), similar to those in the previous iteration, while 1st scan speed (or 2nd scan speed) was fixed at 787 mm s^−1^ (or 1270 mm s^−1^). The predicted initial discharge capacities of these candidates were higher than 90%, but their measured values were lower than 80%. By comparing samples S2 and S25 (or S8 and S26), we can infer that the 2nd scan speed can be one of the reasons for the lower performance.

Figure 2c,d present the regression results of the initial discharge capacity as functions of the 1st scan speed and the 1st laser power, respectively, based on models trained using the final 26 experimental data points, where the 1st scan speed spans a wide range from approximately 100 to 800 mm s^−^^1^ (e.g., 114, 292, 305, 762, 787, and 800 mm s^−^^1^) and the 1st laser power ranges from 1.7 to 4.6 W, with each variable treated as an independent axis for visualization. The MLE Matern model (Figure 2c) and the MLE RBF model (Figure 2d) were employed, while the remaining four input variables were fixed to the conditions of sample S7. Both models exhibit a similar peak in the initial discharge capacity within the first scan speed range of 700–900 mm s^−1^. However, their responses to the 1st laser power differ. In the MLE Matern model, the variation in initial discharge capacity with respect to the 1st laser power is minimal, whereas in the MLE RBF model, a more pronounced dependence on laser power is observed. This indicates that even when using the same dataset, the choice of kernel can lead to different interpretations of the regression surface and optimal conditions. Figure S1 shows the regression results obtained by sequentially adding experimental data from the initial stage up to the third iteration when training the MLE Matern model. As more data are incorporated, the initially near‐constant surface gradually deforms. By the third iteration, a distinct structure emerges around the first scan speed range of 760–790 mm s^−1^, reflecting the concentration of experimental points in this region during the third iteration. The measured values in the fourth iteration were lower than the model predictions, and these data were therefore necessarily included to further refine the regression model. As shown in Figure 2c, incorporating up to the fourth experiment enables a more detailed mapping of the response near the optimal region. Meanwhile, since the proposed models already suggest highly similar optimal input conditions and the candidate values have effectively reached saturation (Figure 2b), further iterative training within the same design space is likely to yield only marginal performance improvement while increasing the experimental cost.

We analyzed the EI values from all GPR models and plotted the mean of the EI values as a function of the optimization iteration in Figure 2e. The first optimization iteration shows the lowest mean EI value; it then increases abruptly in the second optimization iteration and subsequently decreases in the third and fourth optimization iterations. This trend reflects the improved evolution of model uncertainty and the exploration‐exploitation balance: the initial spike in EI at the 1st iteration indicates expanded exploration due to increased model uncertainty with limited training data, while the subsequent decrease suggests that the accuracy of the model is improved toward high‐performance regions with reduced uncertainty. This is consistent with the optimization results shown in Figure 2a, where high‐performance candidates such as S15 and S20 are identified and validated in later iterations. In addition, the PI was also calculated. The PI values also show an increase at the first optimization iteration and a decreasing trend in the second and third optimization iterations, coherently indicating that the accuracy of the GPR models has improved. Considering the behavior in both experimental results (Figure 2a) and acquisition function analysis (Figure 2e), we terminate the EAAL process at the 4th iteration. Overall, this process demonstrates that, as the EAAL iterations progress, the prediction accuracy improves toward the local optimal point, and the quality of the candidate groups improves simultaneously.

Additionally, we compared the accuracy of GPR with commonly used machine learning models such as support vector regression (SVR) [42], and kernel ridge regression (KRR) [43]. These kernel‐based models are frequently employed as strong baselines for regression on small datasets due to their robustness and regularization capabilities [44]. As the twenty‐six data points represent only a tiny fraction of the parameter space (∼ 10^7^ points in total), we employed leave‐one‐out cross‐validation (LOOCV) [45] to obtain a robust estimate of the generalization error. As regression metrics, root mean squared error (RMSE), mean absolute error (MAE), weighted absolute percentage error (WAPE), and mean bias error (MBE), as summarized in Figure S2.

The final error is the average error across the twenty‐six LOOCV validation points, which are shown in Figure S2. Among the compared models, the MLE Matern trained exhibited the best overall performance, achieving the lowest RMSE (0.494) and MAE (0.370). It also showed the smallest WAPE (88.1%), indicating the highest relative accuracy in reproducing the experimental responses. The other GPR‐based models, including MLE RBF and the two MAP‐based models, as well as the kernel‐based baselines SVR and KRR, yielded higher RMSE, MAE, and WAPE values (e.g., WAPE ≥ 95.4%), indicating less accurate predictions compared to MLE Matern. In terms of systematic bias (MBE), all models showed moderate negative bias, with MLE Matern (−0.164) and KRR (−0.098) being among the least biased within this group. Taken together, these results indicate that GPR‐based models outperform the alternative kernel regression approaches considered here, with the MLE Matern configuration providing the most accurate and consistent regression performance for the given small dataset. It should be noted that the relatively high WAPE is a structurally conservative estimate arising from LOOCV on a sparse 26‐point dataset. In this context, predicting isolated extremes without local data generates large absolute errors that disproportionately increase the aggregated the aggregated percentage error; the absolute‐error metrics (RMSE, MAE, MBE) are therefore more appropriate accuracy indicators here and remain small. Moreover, the surrogate used for candidate selection is trained on all available data and is thus more accurate than the LOOCV estimate. The reliability of the surrogate in the decision‐relevant region was confirmed experimentally at the validation points P1–P6 (Figure 4), where the measured capacities lay within the predictive uncertainty bands with residuals of only a few percent.

To evaluate the optimization performance at a trajectory level, trajectory comparison experiments for various optimization strategies were conducted to verify the optimization capability of the EAAL proposed in this study. Given the difficulty of repeated execution in real experimental environments and the necessity of repeated evaluation, a virtual black‐box oracle model was constructed using random forest [46] and KRR to capture the nonlinear input–output relationships and the associated uncertainties of the process. The predictions of the two models were averaged with equal weights to form a single response surface, and a Gaussian noise term was added to reflect measurement variability and uncertainty, thereby constructing a virtual experimental environment.

Based on the 26 training data points used in this study, each experiment was initialized with the same set of five initial data points, and optimization was performed over four iterations, with six experimental conditions selected in each batch, resulting in a total of 24 experimental conditions. In addition, to evaluate the stability and reproducibility of the proposed strategy, the same experimental setup was repeated for five trials using different initial datasets. First, random search (RS) [47] was implemented as a purely random search strategy, in which six experimental conditions were randomly selected from a uniform distribution over the input space at each iteration, and the trajectory was updated based on the best observed value up to that point. Space‐filling Latin hypercube sampling (LHS) [48] was implemented in a manner similar to RS, without model‐based optimization, by generating 24 experimental conditions in advance using a Latin hypercube design and then distributing them in batches of six across four iterations, thereby filling the input space more uniformly and minimizing repetition. BO and the expert‐aided BO were both conducted using the MLE Matern, which showed the best surrogate performance among the tested models, while the empirical‐aided BO further applied the expert‐aided filter to the single MLE Matern. As detailed in the variable dataset selection criteria (Methods 4.2, specifically Rule 2), the expert feasibility filter targets and removes physically unstable combinations of parameter extremes (the upper and lower 10% bounds). Examples include a slow scan speed (<10%) combined with a high pass number (>90%), or a high laser power (>90%) combined with a high pass number (>90%). These specific intersections represent conditions that physically lead to severe thermal degradation, photothermal overdosing, or insufficient energy dosing. To further demonstrate the quantitative necessity of this filter, a detailed comparison of the theoretical and empirically observed infeasible‐proposal rates for each unscreened strategy is provided in the Supporting Information (Table S3).

The corresponding results are presented in Figure 2f, which shows the best observed outputs for five optimization strategies over four batch selection iterations. Each curve represents the mean of the best observed outputs, averaged over five independent runs. The proposed EAAL strategy achieved the highest mean performance, outperforming Random Search, space‐filling LHS, pure BO, and single‐kernel EAAL. This demonstrates the effectiveness of multi‐surrogate ensemble exploration combined with expert‐guided filtering. Notably, the expert‐filtered BO consistently identified better candidate points than pure BO without filtering, confirming the benefit of incorporating domain knowledge into the acquisition process. Notably, the expert‐filtered BO consistently identified better candidate points than pure BO without filtering, confirming the benefit of incorporating domain knowledge into the acquisition process. However, both BO‐based strategies showed performance saturation by the 4^th^ iteration, falling below LHS at the final round. This is likely attributed to the nature of the virtual oracle, which was constructed from the existing training dataset; as the dataset already contains near‐optimal points, the surrogate landscape offers limited room for further improvement, leading to early convergence of the BO strategies. Nevertheless, under this conservative oracle environment—where the oracle itself is built upon an RBF kernel‐based KRR and a random forest, both structurally disadvantageous to GPR—the EAAL framework still identified superior processing conditions compared to non‐adaptive baselines, supporting the robustness of the proposed approach. It should be noted that since the virtual oracles rely on the existing dataset evaluated along the EAAL trajectory, this analysis functions primarily as a retrospective consistency check. It serves to verify that the EAAL trajectory efficiently navigated toward the high‐performing region across different algorithmic environments.

### 2.3 Permutation Importance and Evaluation of Predicted Values

On the other hand, we quantitatively analyzed the correlation between input variables and outputs using the GPR models, which could not be realized by the conventional empirical approach. The permutation importance method (Figure 3a) quantifies the degree to which the model output fluctuates when a specific input variable is perturbed, thereby identifying the importance of each input variable on the output [49]. This variable importance method may fail to provide interpretability if the regression model is not properly trained. Therefore, applying this method to all models may not be appropriate. As shown in Figure 3b, we selected the best‐performing model for further analysis: the MLE Matern model, which demonstrated better prediction accuracy compared to other models as shown in Figure 2f. To quantify variable importance, we calculated permutation importance using the R^2^ score drop. Specifically, the input variables were randomly permuted 30 times, and the decrease in R^2^ score was used as the permutation importance metric for each input variable.

(1) $$R^{2}=1-\frac{\sum_{n}^{N}=1{\left(y_{n}-{\hat{y}}_{n}\right)}^{2}}{\sum_{n}^{N}=1{\left(y_{n}-{\hat{y}}_{n}\right)}^{2}}$$

**FIGURE 3:**
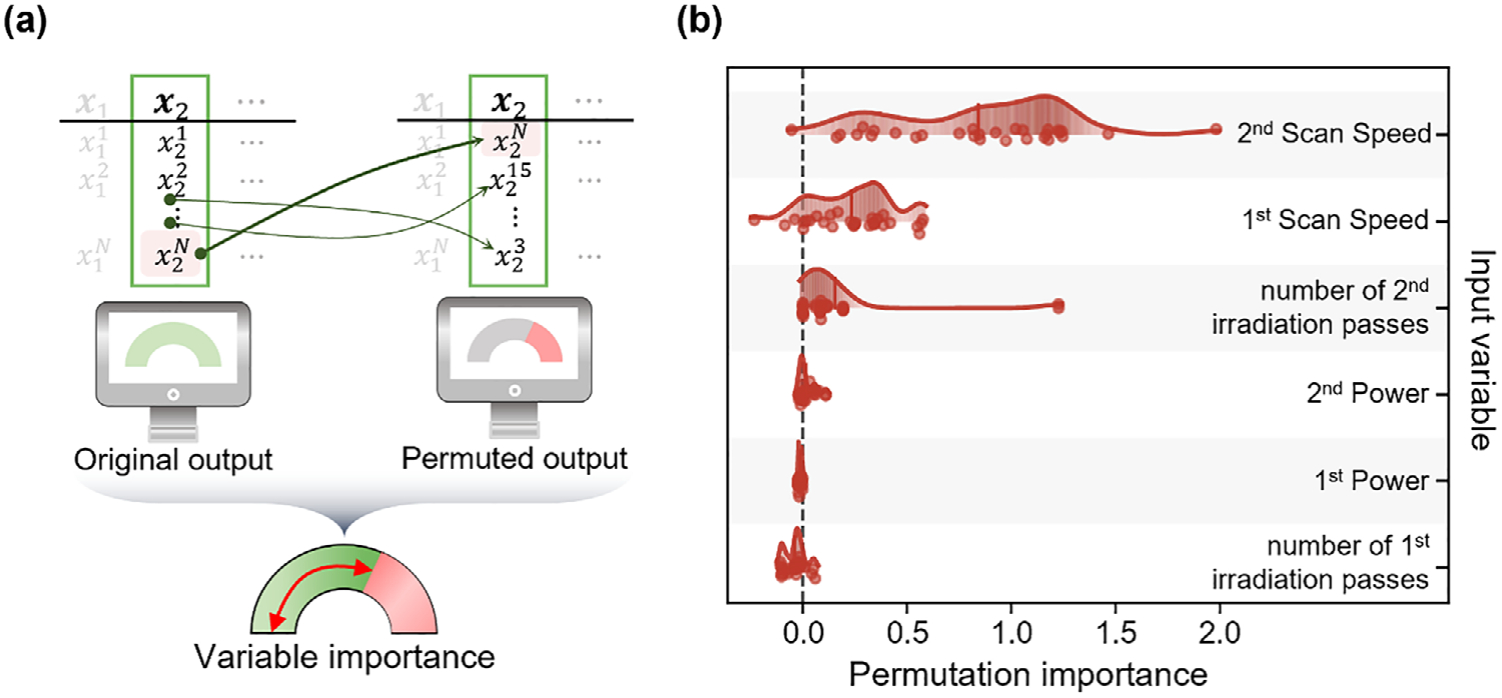
Quantitative relationships between input variables and output performance | (a) Schematic illustration of permutation importance (see main text for details). (b) Variable importance of the six input variables for the initial discharge capacity, obtained from permutation importance.

Here, ${\hat{y}}_{n}$ and *y_n_* represent the actual value and the model predicted value, respectively, and ${\bar{y}}_{n}$ denotes the mean of the actual values. The permutation importance of an input variable is close to 1, which is interpreted as this input having the most significant influence on the 1st discharge rate. The results are presented in Figure 3b. For 2nd scan speed showed mean importance scores of 0.843. It well represents the optimization results in Figure 2a,b. The second most important variable was 1st scan speed, with mean scores of 0.234. While these values are lower than those for 2nd scan speed, they stand in contrast to the lower‐ranked variables such as 2nd laser power, 1st laser power, and number of 1st irradiation passes.

To visualize the input–output relationship, we examined the parameter space near sample S7, which shows the best performance in the EAAL optimization (i.e., sample S7 can alternatively be referred to as the EAAL‐optimized anode). We further investigated the parameter space with experimental validation. Based on the permutation importance analysis, we characterized the parameter space of the GPR models using the two most important input variables: 1st scan speed and 2nd scan speed. Figure 4a shows the predicted initial discharge capacity as a function of 1st scan speed and 2nd scan speed for the MLE Matern GPR model. The analysis was conducted around S7, where the ranges were set to 970–1270 mm s^−1^ for 2nd scan speed and 650–900 mm s^−1^ for 1st scan speed. MLE Matern model identifies six validation points (P1–P6) near S7 (see Table 1). Specifically, P1–P3 were swept at the scan speed 2, P5–P6 at the 1st scan speed, and P4 represents a distinct reference point. We experimentally validated the predicted initial discharge capacities at these points.

**FIGURE 4:**
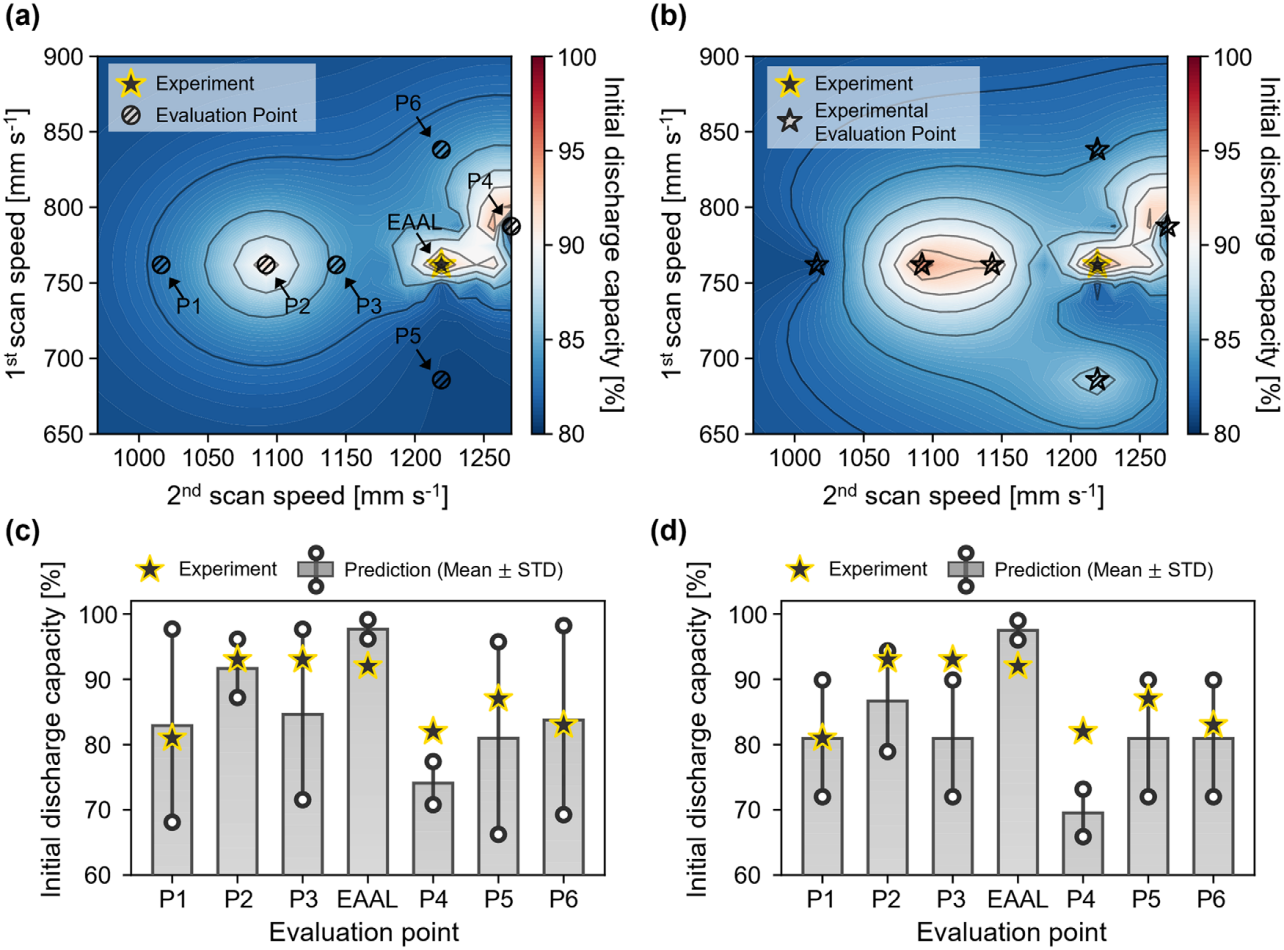
Validation of the parameter space formulated by the GPR models | (a) Predicted initial discharge capacity as a function of 1st scan speed and 2nd scan speed, obtained from the MLE Matern model using twenty‐six samples. The black lines show contours of constant initial discharge capacity. The points marked as ‘P’ indicate the evaluated locations. The star marker indicates S7, which corresponds to the EAAL‐optimized anode (denoted as EAAL in the figure). (b) Refined initial discharge capacity surface as a function of 1st scan speed and 2nd scan speed after including the experimental measurements at the indicated points (‘P1 – P6’). (c,d) The mean and standard deviation (STD) of the predicted and measured initial discharge capacity at the selected points for the (c) MLE Matern and (d) MLE RBF models.

**TABLE 1:** The 1st/2nd laser scan speed conditions in seven evaluation points (S7 and P1–P6).

Evaluation Points	1st Laser Scan Speed [mm s^−1^]	2nd Laser Scan Speed [mm s^−1^]
S7 (= EAAL‐optimized anode)	762	1220
P1	762	1016
P2	762	1092
P3	762	1143
P4	787	1270
P5	686	1220
P6	838	1220

Figure 4b shows the parameter landscape after refining the models by incorporating the experimental data from these locations (P1–P6). The presence of the predicted peak and valleys in previously uncertain regions is confirmed by the added measurements. Interestingly, the spatial distribution of the experimental initial discharge capacities is very similar to the predicted pattern, although the measured values are slightly higher than the predicted initial discharge capacities. Figure 4c,d directly compare the actual experimental values with the predicted values of the models trained without including these validation data. The experimental values fall within the predictive uncertainty range of the models. In addition, the MLE Matern showed smaller overall residuals than the MLE RBF, with residuals ranging from −8.40 to 5.66% compared with −12.5 to 5.50% for the RBF kernel, indicating better agreement with the experimental values in this dataset. Therefore, it is reasonable to conclude that the relationship between the input variables and the initial discharge capacity can be quantified using the GPR models. These results suggest that the EAAL framework is effective for exploring and understanding the parameter space of the initial discharge capacity, even under limited data conditions.

### 2.4 Electrochemical Characteristics and Analysis of the EAAL‐Optimized Anode

To elucidate the physicochemical basis of the experimentally validated parameter space in Figure 4, detailed electrochemical analyses were performed on the corresponding evaluation points (see Figure 5). To investigate the effect of the 2nd laser scan speed on the electrochemical performance, x‐ray photoelectron spectroscopy (XPS) Si 2p spectra were analyzed for the EAAL‐optimized anode and P1–P4 samples, where the 1st laser scan speed is fixed at 760–787 mm s^−1^ and the 2nd laser scan speed is varied from 1143–1220 mm s^−1^ (Figure 5a,b). The spectra were deconvoluted into four subpeaks positioned at 104.0, 103.0, 102.2, and 101.0 eV, respectively [33, 50] (see Figure S3). At the highest 2nd laser scan speed (P4), the fraction of low‐valence Si species (Si^+^ and Si^2+^) remains limited to 8.1 at%, accompanied by a high O/Si ratio of 1.44. These values indicate incomplete carbothermal reduction under limited photothermal energy input, consistent with the reduced initial discharge capacity in Figure 4c,d. As the 2nd laser scan speed decreases from 1220 mm s^−1^ (for the EAAL‐optimized anode) to 1092 mm s^−1^ (for P2), the fraction of low‐valence Si species increases from 39.7 at% to 42.4 at%, while the O/Si ratio decreases from 1.26 to 1.23, confirming the progressive enhancement of carbothermal reduction with increasing energy dose. However, further decrease in scan speed deviates from this trend at P1. The fraction of Si^4+^ increases to 14.8 at% and the O/Si ratio increases to 1.28. This behavior suggests a disproportionation reaction under excessive thermal input, followed by partial surface re‐oxidation of SiO_x_ [51, 52, 53]. These results define a bounded reduction window governed by the 2nd laser scan speed. Insufficient photothermal energy results in incomplete reduction, whereas excessive energy induces surface re‐oxidation that modifies the chemical stoichiometry of SiO_x_. The SiO_x_ anodes with intermediate laser scan speed (P2, P3, and the EAAL‐optimized anode) exhibit an optimized Si stoichiometric distribution characterized by a maximized fraction of low‐valence Si species and moderated Si^4+^ content, corresponding to the peak region predicted in the parameter landscape shown in Figure 4.

**FIGURE 5:**
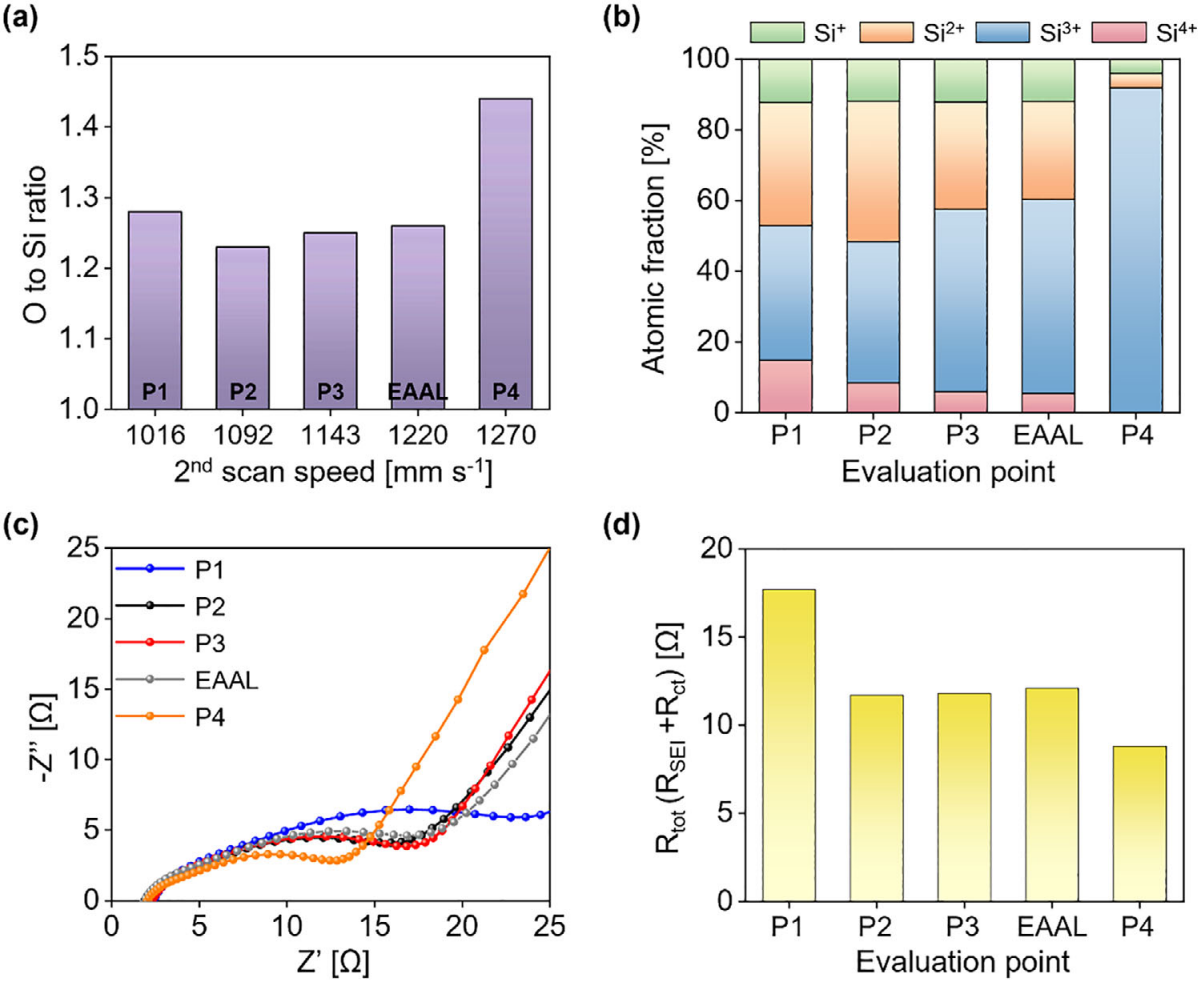
Comprehensive electrochemical evaluation of the EAAL‐optimized anode (i.e., S7, denoted as EAAL in the figure) and P1–P4 anodes | (a) O/Si ratios derived from the XPS Si 2p spectra. (b) Summarized atomic fractions of Si oxidation states. (c) Nyquist plots obtained from assembled half‐cells. (d) Total interfacial resistance (R_tot_ = R_SEI_ + R_ct_) obtained from the Nyquist plots.

To examine the interfacial resistance behavior, electrochemical impedance spectroscopy (EIS) was performed for the same evaluation points (Figure 5c,d). The equivalent circuit model used for fitting is shown in Figure S4. The disproportionated condition, P1, exhibits the highest overall interfacial resistance (R_tot_) of 17.7 Ω, consistent with the elevated Si^4+^ fraction and higher O/Si ratio due to excessive thermal input. The increased surface oxidation is expected to contribute to the formation of a carbonate‐based thick SEI during lithiation and to impede charge‐transfer kinetics [54, 55]. In contrast, P4 with limited thermal input shows a lower R_tot_ of 8.8 Ω, reflecting limited surface SiO_2_‐like oxide formation and thus suppressed interfacial resistance. However, the insufficient carbothermal reduction under this condition results in a limited population of electrochemically active low‐valence Si, accounting for the reduced discharge capacity. The evaluation points with intermediate 2nd laser scan speed (P2, P3, and the EAAL‐optimized anode) display stable impedance responses, indicating suppression of excessive SEI growth while maintaining favorable charge‐transfer kinetics within this bounded processing window. In agreement with the XPS results, these observations confirm that an optimal laser annealing condition enables sufficient formation of electrochemically active low‐valence Si species while simultaneously limiting surface re‐oxidation. Such a balanced stoichiometric state preserves high reversible capacity by providing abundant lithiation sites without promoting excessive interfacial resistance.

A comparable tendency is also observed when the 2nd laser scan speed is fixed at 1220 mm s^−1^ and the 1st scan speed is varied from 686 to 838 mm s^−1^ (Figures S5–S7). Although the 2nd laser scan speed governs the primary carbothermal reduction, the 1st laser scan speed modulates binder carbonization/graphitization and subsequently alters the conductive graphitized network within the electrode [56]. Consequently, the 1st laser scan speed also influences the degree of carbothermal reduction, thereby determining the discharge capacity. This is because the spatial distribution of thermal energy during the 2nd laser scan is affected by the electrically/thermally conductive carbon network produced in the 1st laser scan. At the lowest 1st laser scan speed, P5, localized heat accumulation during the 1st laser scan likely induces excessive carbon restructuring, but it also generates undesirable defects that disrupt highly efficient heat propagation during the 2nd laser scan. Conversely, at the highest 1st laser scan speed, P6 may result in insufficient carbonization, which leads to incomplete thermal propagation in the subsequent 2nd laser scan. The EAAL‐optimized anode, with the intermediate 1st laser scan speed, therefore represents a balanced state in which binder carbonization and defect generation are appropriately tuned. Accordingly, the EAAL‐optimized anode exhibits a higher fraction of electrochemically active low‐valence species with a lower O/Si ratio than P5 and P6, indicating a more effective carbothermal reduction through a defect‐less carbon scaffold without excessive surface re‐oxidation.

To benchmark the electrochemical performance achieved via machine learning, the EAAL‐optimized anode was compared with the ETAE‐optimized anode (Figure 6a). The EAAL‐optimized anode achieves 91.9% of the initial discharge capacity and 96.3% of the initial Coulombic efficiency of the ETAE‐optimized anode at a current density of 0.1 A g^−1^. Such benchmarked values in the EAAL‐optimized anode (in terms of initial discharge capacity and Coulombic efficiency) suggest that the degree of carbothermal reduction, generating electrochemically active low‐valence Si species, is almost comparable to that of the ETAE‐optimized anode (Figures S8 and S9). As previously discussed, the EAAL‐optimized anode resides within the optimal processing‐condition regime, where both carbothermal reduction and surface re‐oxidation reactions are balanced. An abundance of low‐valence Si states ensures sufficient reversible lithiation sites while minimizing unwanted irreversible side reactions (excessive solid electrolyte interphase growth). Furthermore, the laser‐induced photothermal processing promotes internal mesopore formation within the SiO_x_ matrix, which mitigates mechanical stress associated with volume expansion and contributes to sustained cycling stability (Figures S10 and S11). Accordingly, the EAAL‐optimized anode fully maintains the long‐term cycling stability of the ETAE‐optimized anode at a current density of 0.5 A g^−1^, demonstrating that structural robustness is largely preserved even under the reduced‐iteration history.

**FIGURE 6:**
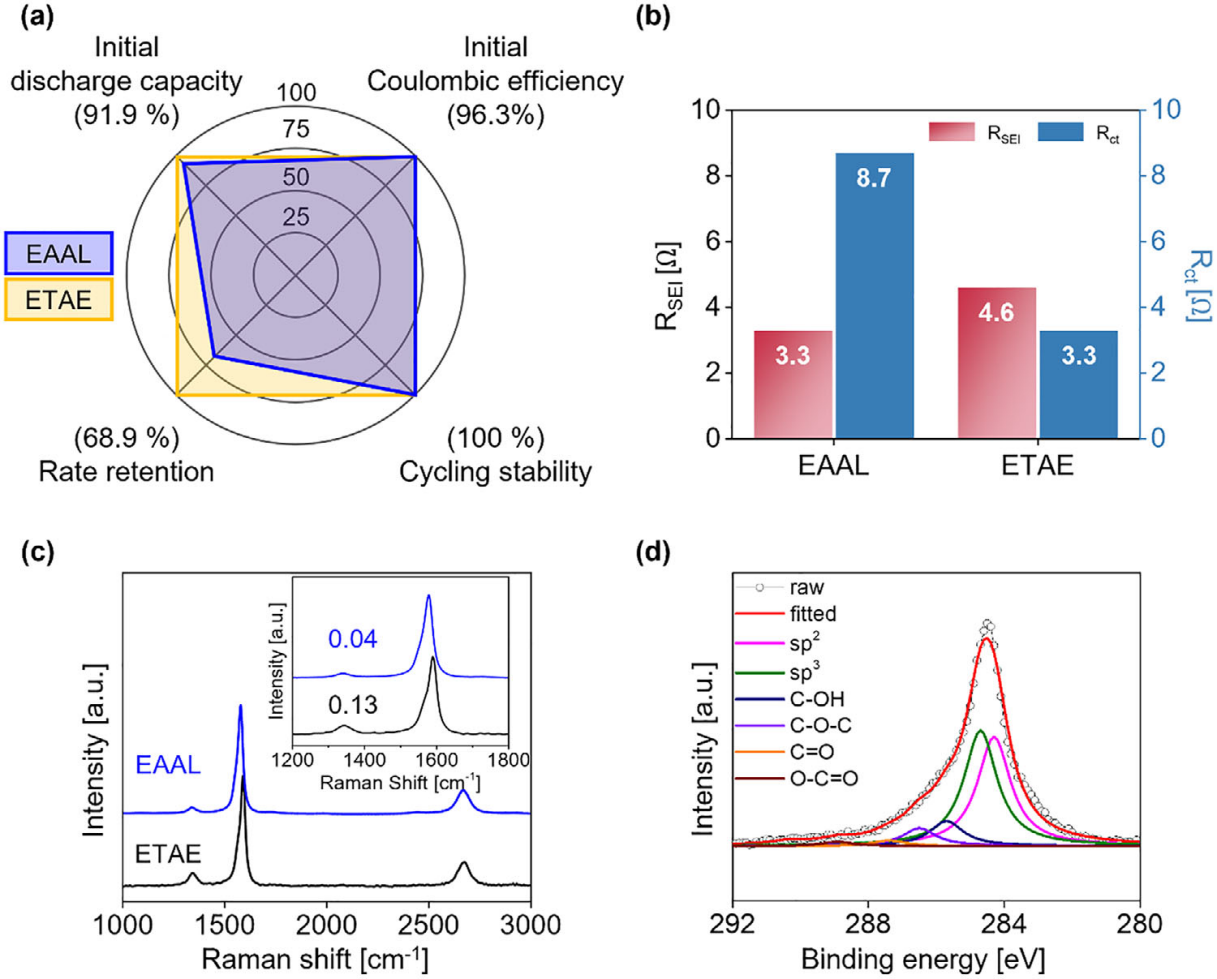
Performance comparison between the EAAL‐ and ETAE‐optimized anodes (denoted as EAAL and ETAE in the figure, respectively) | (a–c) (a) Radar charts comparing the overall performance metrics, (b) R_SEI_ and R_ct_ obtained from the Nyquist plots, and (c) Raman spectroscopy spectra of both anodes. (d) XPS C 1s spectrum of the EAAL‐optimized anode.

To compare the interfacial characteristics between the EAAL‐ and ETAE‐ optimized anodes, EIS spectra were comparatively analyzed (Figure 6b and Figure S12). Deconvolution yields R_SEI_ and R_ct_ values of 3.3 and 8.7 Ω for the EAAL‐optimized anode, and 4.6 and 3.3 Ω for the ETAE‐optimized anode, respectively. The EAAL‐optimized anode exhibits a lower R_SEI_, suggesting that limiting cumulative irradiation suppresses progressive surface re‐oxidation and mitigates excessive solid electrolyte interphase growth. In contrast, the higher R_ct_ correlates with the reduced rate capability at a current density of 2.0 A g^−1^ (68.9% retention), indicating slower interfacial charge‐transfer kinetics relative to the ETAE‐optimized anode (Figure S13). To probe the origin of this kinetic difference, Raman spectroscopy was conducted (Figure 6c).  The characteristic peaks of the D‐ and G‐bands are detected at 1350 and 1590 cm^−1^, respectively, corresponding to disordered and graphitic carbons [57]. The EAAL‐optimized anode exhibits a lower I_D_/I_G_ (0.04) than the ETAE‐optimized anode (0.13), reflecting more pronounced binder carbonization/graphitization. This implies that the graphitized carbons are partially degraded into defective structures by the five‐pass laser irradiation in the ETAE‐optimized anode. Although well‐developed graphitized carbon networks enhance electronic conduction pathways, they may simultaneously increase surface hydrophobicity and reduce electrolyte wettability, thereby elevating charge‐transfer resistance [58, 59]. To further confirm this, we also analyzed XPS C 1s spectra (Figure 6d and Figure S14). The C 1s spectrum is deconvoluted into six subpeaks, corresponding to the C─C (sp^2^), C─C (sp^3^), C─OH, C═O, and O─C═O bonds, located at 284.3, 284.7, 285.7, 286.5, 287.4, and 288.9 eV, respectively [60]. The ratio of C‐C (sp^2^) to C‐C (sp^3^) increases to 48.5% in the EAAL‐optimized anode, compared with the value of 25.7% for the ETAE‐optimized anode, which is in line with the interpretation from the Raman spectra for both anodes.

## 3 Conclusion

In summary, we developed an EAAL framework to efficiently optimize laser‐induced photothermal annealing for SiO_x_ anodes. By synergistically integrating probabilistic machine learning with empirical domain knowledge, the EAAL framework effectively navigates a complex processing parameter space using a limited number of experiments. Through iterative optimization cycles, the EAAL framework rapidly converges toward the optimal region within only four iterations using 26 experimental data points. Systematic analysis of multiple GPR models reveals that kernel selection and hyperparameter optimization critically influence the prediction of the parameter–performance landscape, while sensitivity analysis identifies the 1st and 2nd scan speeds as dominant variables governing the electrochemical response. Experimental validation combined with electrochemical and spectroscopic analyses confirms that an optimal processing window exists, where carbothermal reduction and surface re‐oxidation are balanced to maximize the fraction of electrochemically active low‐valence Si species while maintaining structural stability. Within this optimized regime, post‐optimization electrochemical validation confirmed that the EAAL‐optimized anode achieves 91.9% of the initial discharge capacity and 96.3% of the initial Coulombic efficiency of the ETAE‐optimized counterpart. Importantly, this comparable performance was achieved while significantly reducing the number of laser irradiation passes from five to two, thereby improving process efficiency. Furthermore, the EAAL‐optimized anode fully retains the long‐term cycling stability of the ETAE‐optimized anode. These results demonstrate that the EAAL framework provides a data‐efficient and physically interpretable strategy for process optimization, highlighting the importance of incorporating empirical knowledge into machine learning‐assisted materials design and offering a scalable pathway toward advanced manufacturing of energy storage systems.

## 4 Methods

### 4.1 Gaussian Process Regression With Kernel Functions

GPR was employed as a surrogate model to describe the relationship between the input variables and the target responses. Among various kernel functions, the RBF kernel is defined as:

(2) $$k_{RBY}\left(x,x^{\prime }\right)=\sigma_{f}^{2}\exp\left(\frac{{\left(x,x^{\prime }\right)}^{2}}{2\ell^{2}}\right)$$

where *x* and $\;x^{\prime }$ represent distinct data point inputs, with the hyperparameter ℓ defining the length scale. Utilizing the RBF kernel implies that the surrogate model is infinitely differentiable, resulting in the generation of a significantly smooth function. The length scale, a parameter of the kernel, determines the input width of the kernel and the degree of smoothness. We used anisotropic length scales for the input variables, meaning each variable has its own length scale. For example, a small (or larger) length scale for an input variable means that the model output varies sensitively (or insensitively) with changes in the input variable. The Matern kernel is given by [61] in:

(3) $$\begin{array}{ccc} & & k_{Matern}\left(x,x^{\prime }\right)\\ & & =\frac{2^{1-v}}{\Gamma \left(v\right)}\;{\left(\sqrt{\frac{2v}{2\ell^{2}}{\left(x-x^{\prime }\right)}^{2}}\right)}^{v}k_{v}\left(\sqrt{\frac{2v}{2\ell^{2}}{\left(x-x^{\prime }\right)}^{2}}\right) \end{array}$$

with hyperparameter ν and ℓ, $\kappa_{\nu }$ is a modified Bessel function [62]. While the RBF kernel assumes only infinitely smooth functions, the Matern kernel allows for the adjustment of the degree of smoothness using hyperparameters. When ν→∞, the Matern kernel functions identically to the RBF kernel. The Matern kernel forms a less smooth surrogate model, thereby modeling a different objective function compared to the RBF kernel, even when using identical data.

### 4.2 Variable Dataset Selection and Candidate Selection Policy

The selection criteria for the variable dataset are critical not only for constructing the initial training set but also for guiding the subsequent selection of candidate conditions during the EAAL iterations. We therefore distinguish two stages: (a) the construction of the initial training dataset, and (b) the candidate selection policy applied at each subsequent EAAL iteration.

**(a) Initial training dataset construction**. Rather than relying on a purely ad hoc choice of initial conditions, we adopted a structured strategy that combines space‐filling design principles with domain knowledge. First, candidate conditions were generated to broadly cover the processing window and to ensure sufficient variation in each input variable. Conditions that were physically unrealistic or practically inefficient (e.g., combinations leading to excessive processing time, severe thermal damage, or negligible energy input) were then excluded based on empirical criteria. Specifically, three types of data combinations were discarded: (i) conditions combining a low scan speed with a high number of irradiation passes, which result in unreasonably long processing time; (ii) conditions combining high laser power with a high number of passes, which carry a high risk of thermal degradation and non‐uniform electrode surfaces; and (iii) conditions combining low laser power with high scan speed, where the energy dose is too low to induce meaningful thermal modification of the sample. Finally, among the remaining feasible conditions, we selected a subset that maximized the overall spread in the input space, thereby providing a diverse and informative starting dataset for the EAAL workflow.

**(b) Candidate selection policy during EAAL iterations**. At each iteration, the four GPR models (RBF/Matern × MLE/MAP) each propose three candidates, yielding twelve model‐suggested candidates, from which six are selected for experimental verification. This reduction is governed by an explicit, prioritized policy applied in the order Rule 1 → Rule 2 → Rule 3:

**Rule 1 (Mandatory acquisition‐driven inclusion; four candidates)**. For each of the four models, the single candidate with the highest EI value is mandatorily selected, independent of expert judgment. This guarantees that four of the six selected candidates per iteration are dictated solely by the acquisition policy. In the fourth iteration, as the optimization approached saturation, an adaptive PI criterion was additionally considered alongside EI to prioritize exploitation (see the adaptive trade‐off parameter ξ_adaptive defined below).
**Rule 2 (Deterministic feasibility exclusion)**. Candidates are strictly excluded if they fall into one of the three physically prohibited coupled‐variable regimes defined in (a)—namely (i) low scan speed with a high number of passes, (ii) high laser power with low scan speed or a high number of passes, and (iii) low laser power with high scan speed. These criteria define the feasible region used consistently throughout this work.
**Rule 3 (Expert selection of the remaining two candidates)**. From the eight remaining candidates (the twelve model‐suggested candidates minus the four mandatory top‐EI points), domain experts select two, based on the feasibility criteria of Rule 2 and on process‐parameter diversity—for example, broadening laser‐power coverage, probing boundary conditions such as the highest scan speed, or testing alternative irradiation‐pass counts. This step is the only one involving expert discretion, and it operates strictly within the feasible set defined by Rules 1,2, bounding expert influence to two of the six candidates (one‐third of each batch). The unselected candidates in this step are rejected due to lower priority.

The complete evaluation log for all twelve model‐suggested candidates at each iteration—including the predicted mean (µ), predictive standard deviation (σ), acquisition values (EI/PI), the selected model, and the acceptance/rejection decision together with its physical justification—is provided in Table S1, allowing the full optimization trajectory to be reproduced.

During the EAAL iterations, candidate prioritization was guided by EI. From the 1st to the 3rd iterations, the candidate exhibiting the highest EI value from each model was selected as a mandatory inclusion for experimental verification. However, in the 4th iteration, an adaptive PI was additionally employed to maximize exploitation. To suppress unguided exploration in highly uncertain regions, an adaptive trade‐off parameter, ξ_*adaptive*_, was introducted into PI formulation:

(4) $$\xi_{adaptive}=\xi_{base}\times \left(\frac{\sigma }{\sigma \max}\right)$$

where ξ_*base*_ is set to 0.05, and σ_*max*_ is the maximum standard deviation among candidates. By ξ_*adaptive*_ proportional to σ, this mechanism penalizes unexplored domains, ensuring that the algorithm confidently recommends candidates from reliably modeled optimal regions.

### 4.3 Computational Details

MLE GPR models, SVR, and KRR models were implemented in Python using the scikit‐learn library, whereas MAP GPR models were implemented using PyTorch and Pyro. Model evaluation under LOOCV was performed with a fixed global random seed of 40 applied consistently across NumPy, PyTorch, and Pyro to ensure reproducibility of the regression metrics. Hyperparameters for all surrogate models were tuned using simple, model‐appropriate procedures. For SVR and KRR, we employed RBF kernels and performed small grid searches over representative values of the regularization strength and kernel width using GridSearchCV with 6‐fold cross‐validation on the training data. For the GPR models with RBF and Matern kernels, kernel hyperparameters (length scales and noise level) were optimized by maximizing the MLE or by variational MAP inference in Pyro, starting from reasonable initial values and constraining length scales to a broad range while enforcing non‐negative noise variance. Trajectory comparison experiments for the different optimization strategies were initialized with a distinct random seed (42) for all models, so that each strategy experienced an identical stochastic environment while remaining independent from the LOOCV evaluation settings. All computations were carried out using double‐precision floating‐point numbers on the CPU.

### 4.4 Materials

Vinyltriethoxysilane (VTES), hexadecyltrimethylammonium bromide (CTAB), ammonium hydroxide (NH_4_OH, 30%), polyvinylpyrrolidone (PVP, M.W. 55 000 and 360 000), polyacrylic acid (PAA, M.W. 450 000), and ethylene glycol (EG, 99%) were purchased from Sigma–Aldrich and used without further purification. Graphene oxide (GO) dispersion (1 wt.%) was purchased from Standard Graphene. Super P (Timcal, Switzerland) and single‐walled carbon nanotubes (SWCNTs) dispersion (0.4 wt.%, S1304WA, Betterial Co., Republic of Korea) were purchased and used as‐received. Lithium metal foil and electrolyte consisting of 1.3 m LiPF_6_ in ethylene carbonate (EC)/ethyl methyl carbonate (EMC) (3:7 v/v) and 5 wt.% fluoroethylene carbonate (FEC) were purchased from Welcos.

### 4.5 Preparation of Laser‐Annealed Mesoporous SiO_x_ Anodes

SiO_2_ nanoparticles were synthesized via an oil‐in‐water emulsion‐based sol‐gel method. First, 12.9 g of ethanol and 25.3 g of deionized (DI) water were mixed and stirred for 5 min. 0.08 g of CTAB was then added, followed by an additional 5 min of stirring. Subsequently, 0.45 g of VTES was added to the mixture. 0.45 g of NH_4_OH and 25 mg of PVP (M.W. 55 000) were added, and the mixture was allowed to react for 3 h to complete the sol‐gel process. 300 mg of the as‐prepared SiO_2_ nanoparticles was dispersed in a mixture containing 30 mg of PVP (M.W. 360 000), 200 g of DI water, and 7.5 g of 1 wt.% GO dispersion. The resulting particles were collected by centrifugation, dried, and thermally annealed at 800°C for 3 h under an argon atmosphere. The electrode slurry was prepared in EG with a weight ratio of 7:0.8:0.2:1 for active material, Super P, SWCNTs, and PAA, respectively. The slurry was cast on Cu foil with a total electrode mass loading of 1.0 mg cm^−2^. Laser annealing was carried out using a mid‐infrared CO_2_ laser (wavelength of 10.6 µm). During laser scanning, adjacent scan lines were overlapped with an interval of 12.7 µm.

### 4.6 Material Characterization

The morphology and pore structure of the active materials were observed using transmission electron microscopy (Thermo Fisher Scientific, Talos F200X). Raman spectra were obtained using a Raman spectrometer (FEX, WEVE). XPS measurements were performed with Thermo Scientific K‐Alpha instrument. For postmortem analysis, the electrodes were obtained from the disassembled cells and thoroughly rinsed with dimethyl carbonate to remove residual electrolyte, lithium salts, and surface‐bound organic species.

### 4.7 Electrochemical Characterization

Half‐cells were assembled in CR2032 coin‐type cells using lithium metal foil as the counter electrode and a polypropylene separator (Celgard 2400, MTI, 25 µm, porosity: 41%). 1.3 m LiPF_6_ in EC/EMC (3:7 v/v) with 5 wt.% FEC was used for the electrolyte. Galvanostatic charge‐discharge tests were conducted over a voltage window of 0.01–3 V (vs. Li^+^/Li) using a WBCS3000LE cycler (Wonatech, Korea). All the electrochemical tests were carried out at room temperature. The EIS was performed using an VMP‐3 workstation (BioLogic) to investigate the electrochemical interfacial properties. The measurements were performed over a frequency range from 1 MHz to 10 mHz with an AC amplitude of 10 mV.

## Author Contributions

All authors designed the study, and drafted the manuscript; C.P. performed GPR part and Y.L. performed empirical‐aided optimization part; C.P. and E.L. mainly designed and performed theoretical modeling, machine learning, and optimization; Y.L. and S.J. mainly designed and performed experimental fabrication, characterization, and electrochemical analysis. All authors read and approved the final manuscript.

## Funding

This work was supported by the National Research Foundation of Korea (NRF) grant funded by the Korea government (MSIT) (RS‐2025‐16064483 and NRF‐2023R1A2C2005280). This research was supported by the Institute of Information & communications Technology Planning & Evaluation (IITP) grant (RS‐2024‐00393808, Efficient design of RF components and systems based on artificial intelligence) funded by the Korea government (MSIT).

## Conflicts of Interest

The authors declare no conflicts of interest.

## Supporting information




**Supporting File**: advs76607‐sup‐0001‐SuppMat.docx.

## Data Availability

The data that support the findings of this study are available from the corresponding author upon reasonable request.
