# Supplementary material for: Human‐Guided Bayesian Optimization Enables High‐Throughput Laser Annealing of Mesoporous SiOx Anodes for Lithium‐Ion Batteries
Source: Adv Sci (Weinh). 2026 Jul 17;13(56):e76607. doi: 10.1002/advs.76607 (PMC13379250; doi:10.1002/advs.76607)
Supplement: Supplementary file 1 [file ADVS-13-e76607-s001.docx]

Supporting Information

Human-Guided Bayesian Optimization Enables High-Throughput Laser Annealing of Mesoporous SiO_x_ Anodes for Lithium-Ion Batteries

Chaeyoung Park^1‡^, Yeongje Lee^2‡^, Sunho Jeong^2*^, Eungkyu Lee^1*^

Chaeyoung Park and Prof. Eungkyu Lee

^1^Department of Electronic Engineering, Kyung Hee University, 1732, Deogyeong-daero, Giheung-gu, Yongin-si, Gyeonggi-do, 17104, Republic of Korea

Yeongje Lee and Prof. Sunho Jeong

^2^Department of Materials Science and Engineering, Kyung Hee University, 1732, Deogyeong-daero, Giheung-gu, Yongin-si, Gyeonggi-do, 17104, Republic of Korea

^‡^Chaeyoung Park and Yeongje Lee are eqaully contributed.

*Correspondence: Sunho Jeong ([sjeong@khu.ac.kr](mailto:sjeong@khu.ac.kr)) | Eungkyu Lee ([eleest@khu.ac.kr](mailto:eleest@khu.ac.kr))

**Table S1.** Detailed optimization history of the model suggested candidates per iteration, including process variables, acquisition function values, selected machine learning models, and the decisions with their corresponding justifications.

| Iter. | No. | Input | | | | | | Acquisition function | | Output | | Selected Model | Status (Reason) |  |
| --- | --- | --- | --- | --- | --- | --- | --- | --- | --- | --- | --- | --- | --- | --- |
|  |  | Step 1 | | | Step 2 | | |  |  |  |  |  |  |  |
|  |  | Laser  power  [W] | Scan  speed  [mm s^-1^] | number of  irradiation  passes  [#] | Laser  power  [W] | Scan  speed  [mm s^-1^] | number of  irradiation  passes  [#] | EI | PI | $\mu$  [%] | $\sigma$  [%] |  |  |  |
| 1 | C1 | 4.5 | 114 | 1 | 4.5 | 1270 | 1 | 0.0725 |  | 94.78 | 3.99 | MLE RBF | Accepted  (Rule 1) | |
|  | C2 | 4.5 | 267 | 1 | 4.5 | 1270 | 1 | 0.0705 |  | 95.43 | 3.23 |  | Rejected  (Rule 2) | |
|  | C3 | 4.2 | 64 | 1 | 4.5 | 1270 | 1 | 0.0663 |  | 94.36 | 4.16 |  | Rejected  (Rule 2) | |
|  | C4 | 4.5 | 368 | 1 | 4.5 | 1270 | 1 | 0.0604 |  | 94.49 | 3.8 | MLE Matern | Accepted  (Rule 1) | |
|  | C5 | 4.5 | 254 | 1 | 4.5 | 1270 | 1 | 0.0591 |  | 93.69 | 4.48 |  | Rejected  (Rule 2) | |
|  | C6 | 4.2 | 318 | 1 | 4.5 | 1270 | 1 | 0.0542 |  | 93.96 | 4.03 |  | Accepted  (Rule 3) | |
|  | C7 | 3.9 | 775 | 1 | 4.2 | 1219 | 1 | 0.0788 |  | 93.27 | 5.67 | MAP RBF | Accepted  (Rule 1) | |
|  | C8 | 3.6 | 762 | 1 | 4.2 | 1219 | 1 | 0.077 |  | 91.67 | 6.75 |  | Accepted  (Rule 3) | |
|  | C9 | 4.2 | 749 | 1 | 4.2 | 1219 | 1 | 0.0768 |  | 91.08 | 6.92 |  | Rejected  (Lower priority) | |
|  | C10 | 3.9 | 787 | 1 | 4.2 | 1232 | 1 | 0.0671 |  | 92.99 | 5.31 | MAP Matern | Accepted  (Rule 1) | |
|  | C11 | 4.2 | 749 | 1 | 4.2 | 1232 | 1 | 0.0661 |  | 89.9 | 7.08 |  | Rejected  (Lower priority) | |
|  | C12 | 3.9 | 775 | 1 | 4.2 | 1232 | 1 | 0.0649 |  | 92.54 | 5.68 |  | Rejected  (Lower priority) | |
| 2 | C1 | 4.2 | 749 | 1 | 4.2 | 1219 | 1 | 0.0877 |  | 95.92 | 5.39 | MLE RBF | Accepted  (Rule 1) | |
|  | C2 | 3.9 | 749 | 1 | 4.2 | 1219 | 1 | 0.0877 |  | 96.17 | 5.14 |  | Rejected  (Lower priority) | |
|  | C3 | 3.6 | 749 | 1 | 4.2 | 1219 | 1 | 0.0872 |  | 96.27 | 5.02 |  | Accepted  (Rule 3) | |
|  | C4 | 3.6 | 749 | 1 | 4.2 | 1194 | 1 | 0.0848 |  | 95.15 | 5.99 | MLE Matern | Accepted  (Rule 1) | |
|  | C5 | 3.3 | 762 | 1 | 4.2 | 1232 | 1 | 0.0768 |  | 94.4 | 6.3 |  | Rejected  (Rule 2) | |
|  | C6 | 4.5 | 787 | 1 | 4.2 | 1270 | 1 | 0.0561 |  | 91.37 | 7.64 |  | Rejected  (Rule 3) | |
|  | C7 | 4.2 | 762 | 1 | 4.2 | 1257 | 1 | 0.3528 |  | 100.97 | 8.81 | MAP RBF | Accepted  (Rule 1) | |
|  | C8 | 3.9 | 749 | 1 | 4.2 | 1245 | 1 | 0.352 |  | 100.79 | 9.09 |  | Rejected  (Rule 3) | |
|  | C9 | 3.6 | 749 | 1 | 4.2 | 1245 | 1 | 0.3351 |  | 100.68 | 8.61 |  | Accepted  (Rule 3) | |
|  | C10 | 3.9 | 749 | 1 | 4.2 | 1245 | 1 | 0.2226 |  | 96.67 | 9.6 | MAP Matern | Accepted  (Rule 1) | |
|  | C11 | 3.6 | 749 | 1 | 4.2 | 1245 | 1 | 0.2217 |  | 96.91 | 9.32 |  | Rejected  (Lower priority) | |
|  | C12 | 3.6 | 737 | 1 | 4.2 | 1232 | 1 | 0.2202 |  | 96.27 | 9.94 |  | Rejected  (Lower priority) | |
| 3 | C1 | 4.2 | 762 | 1 | 4.2 | 1219 | 1 | 0.0737 |  | 96.27 | 4.49 | MLE RBF | Accepted  (Rule 1) | |
|  | C2 | 3.9 | 762 | 1 | 4.2 | 1219 | 2 | 0.0728 |  | 97.01 | 3.71 |  | Accepted  (Rule 3) | |
|  | C3 | 4.5 | 787 | 1 | 3.9 | 1270 | 1 | 0.0674 |  | 96.63 | 3.88 |  | Rejected  (Lower priority) | |
|  | C4 | 4.5 | 787 | 4 | 3.6 | 1270 | 3 | 0.0894 |  | 86.4 | 12.97 | MLE Matern | Accepted  (Rule 1) | |
|  | C5 | 4.2 | 787 | 4 | 4.5 | 1270 | 4 | 0.0877 |  | 85.73 | 13.33 |  | Rejected  (Rule 2) | |
|  | C6 | 3.9 | 787 | 5 | 4.5 | 1270 | 3 | 0.0862 |  | 85.41 | 13.47 |  | Rejected  (Rule 2) | |
|  | C7 | 3.3 | 762 | 1 | 4.2 | 1181 | 1 | 0.2205 |  | 98.13 | 7.78 | MAP RBF | Accepted  (Rule 1) | |
|  | C8 | 3.6 | 762 | 1 | 4.2 | 1181 | 1 | 0.2132 |  | 98.17 | 7.52 |  | Rejected  (Lower priority) | |
|  | C9 | 3 | 762 | 1 | 4.2 | 1168 | 1 | 0.2109 |  | 95.98 | 9.99 |  | Rejected  (Rule 2) | |
|  | C10 | 3.3 | 775 | 1 | 4.2 | 1194 | 1 | 0.173 |  | 96.31 | 8.3 | MAP Matern | Accepted  (Rule 1) | |
|  | C11 | 4.5 | 787 | 1 | 4.2 | 1270 | 1 | 0.1664 |  | 96.89 | 7.41 |  | Rejected  (Lower priority) | |
|  | C12 | 4.5 | 800 | 1 | 4.2 | 1270 | 1 | 0.1471 |  | 94.81 | 8.83 |  | Accepted  (Rule 3) | |
| 4 | C1 | 4.5 | 787 | 2 | 3.9 | 1270 | 1 | 0.0815 |  | 96.7 | 4.37 | MLE RBF | Accepted  (Rule 3) | |
|  | C2 | 4.2 | 787 | 2 | 3.6 | 1270 | 1 | 0.0775 |  | 96.51 | 4.4 |  | Rejected  (Lower priority) | |
|  | C3 | 3.9 | 787 | 2 | 3.6 | 1270 | 1 | 0.0729 |  | 95.85 | 4.85 |  | Rejected  (Lower priority) | |
|  | C4 | 4.5 | 851 | 2 | 3.6 | 1270 | 1 | 0.0953 |  | 86.82 | 12.99 | MLE Matern | Rejected  (Lower priority) | |
|  | C5 | 4.2 | 851 | 2 | 3.6 | 1270 | 1 | 0.0952 |  | 86.81 | 12.99 |  | Rejected  (Lower priority) | |
|  | C6 | 3.9 | 864 | 2 | 3.9 | 1270 | 1 | 0.0952 |  | 86.37 | 13.3 |  | Rejected  (Lower priority) | |
|  | C7 | 4.2 | 787 | 3 | 3.6 | 1270 | 1 | 0.1374 |  | 94.68 | 8.58 | MAP RBF | Rejected  (Lower priority) | |
|  | C8 | 3.9 | 787 | 3 | 3.9 | 1270 | 1 | 0.1367 |  | 95.35 | 7.9 |  | Accepted  (Rule 3) | |
|  | C9 | 4.5 | 787 | 3 | 3.6 | 1270 | 1 | 0.1366 |  | 94.54 | 8.67 |  | Accepted  (Rule 3) | |
|  | C10 | 4.5 | 787 | 2 | 3.9 | 1270 | 1 | 0.1266 |  | 96.03 | 6.81 | MAP Matern | Rejected  (Lower priority) | |
|  | C11 | 4.2 | 787 | 3 | 3.9 | 1270 | 1 | 0.1212 |  | 93.94 | 8.6 |  | Rejected  (Lower priority) | |
|  | C12 | 3.9 | 787 | 3 | 3.9 | 1270 | 1 | 0.1153 |  | 93.44 | 8.79 |  | Accepted  (Duplicate of C8) | |
|  | C13 | 4.5 | 787 | 1 | 4.2 | 1270 | 1 |  | 0.3849 | 97.03 | 3.97 | MLE RBF | Accepted  (Rule 3) | |
|  | C14 | 4.2 | 787 | 1 | 4.2 | 1270 | 1 |  | 0.3839 | 97.07 | 3.72 |  | Accepted  (Rule 3) | |
|  | C15 | 3.9 | 787 | 1 | 4.2 | 1270 | 1 |  | 0.3348 | 96.39 | 4.28 |  | Accepted  (Rule 3) | |
|  | C16 | 3.6 | 762 | 1 | 4.2 | 1219 | 1 |  | 0.2892 | 96.86 | 1.98 | MLE Matern | Rejected  (Lower priority) | |
|  | C17 | 4.5 | 787 | 1 | 4.2 | 1270 | 1 |  | 0.2885 | 95.54 | 4.58 |  | Accepted  (Duplicate of C12) | |
|  | C18 | 4.2 | 787 | 1 | 4.2 | 1270 | 1 |  | 0.2869 | 95.53 | 4.57 |  | Accepted  (Duplicate of C13) | |
|  | C19 | 4.5 | 787 | 1 | 4.2 | 1270 | 1 |  | 0.4598 | 99.2 | 3.65 | MAP RBF | Accepted  (Rule 3) | |
|  | C20 | 3.6 | 762 | 1 | 4.2 | 1207 | 1 |  | 0.4462 | 98.88 | 2.79 |  | Rejected  (Lower priority) | |
|  | C21 | 4.2 | 787 | 1 | 4.2 | 1270 | 1 |  | 0.4253 | 98.94 | 2.94 |  | Accepted  (Duplicate of C13) | |
|  | C22 | 4.5 | 787 | 1 | 4.2 | 1270 | 1 |  | 0.4672 | 97.75 | 4.15 | MAP Matern | Accepted  (Duplicate of C12) | |
|  | C23 | 4.2 | 787 | 1 | 4.2 | 1270 | 1 |  | 0.453 | 97.61 | 3.85 |  | Accepted  (Duplicate of C13) | |
|  | C24 | 3.9 | 787 | 1 | 4.2 | 1270 | 1 |  | 0.3898 | 96.81 | 4.67 |  | Accepted  (Duplicate of C14) | |

**Table S2.** Detailed optimization history for the 26 experimental data points. The table summarizes the acquisition values, surrogate model predictions, and experimental outcomes for each candidate. All predicted values are derived from the selected model at each corresponding stage, where $\mu$ and $\sigma$ denote the predicted mean and standard deviation, respectively.

| Iter. | No. | Input | | | | | | Acquisition function | | Output | | Exp. | Selected Model |
| --- | --- | --- | --- | --- | --- | --- | --- | --- | --- | --- | --- | --- | --- |
|  |  | Step 1 | | | Step 2 | | |  |  |  |  |  |  |
|  |  | Laser  power  [W] | Scan  speed  [mm s^-1^] | number of  irradiation  passes  [#] | Laser  power  [W] | Scan  speed  [mm s^-1^] | number of  irradiation  passes  [#] | EI | PI | $\mu$  [%] | $\sigma$  [%] |  |  |
| Initial | #1 | 2.4 | 292 | 2 | 2.4 | 305 | 3 |  |  |  |  | 88 |  |
|  | #2 | 4.2 | 787 | 1 | 4.2 | 1257 | 1 |  |  |  |  | 96 |  |
|  | #3 | 4.2 | 305 | 3 | 2.7 | 559 | 2 |  |  |  |  | 81 |  |
|  | #4 | 1.8 | 864 | 1 | 2.4 | 635 | 2 |  |  |  |  | 84 |  |
|  | #5 | 3 | 762 | 1 | 4.2 | 1092 | 1 |  |  |  |  | 92 |  |
| 1 |  | 4.5 | 114 | 1 | 4.5 | 1270 | 1 | 0.0725 |  | 94.78 | 3.99 | - | MLE  RBF |
|  |  | 4.5 | 368 | 1 | 4.5 | 1270 | 1 | 0.0604 |  | 94.49 | 3.80 |  | MLE Matern |
|  |  | 4.2 | 318 | 1 | 4.5 | 1270 | 1 | 0.0542 |  | 93.96 | 4.03 |  | MLE Matern |
|  | #6 | 3.9 | 775 | 1 | 4.2 | 1219 | 1 | 0.0788 |  | 93.27 | 3.76 | 89 | MAP  RBF |
|  | #7 | 3.6 | 762 | 1 | 4.2 | 1219 | 1 | 0.077 |  | 91.67 | 4.48 | 98 | MAP  RBF |
|  | #8 | 3.9 | 787 | 1 | 4.2 | 1232 | 1 | 0.0671 |  | 93.29 | 3.61 | 83 | MAP Matern |
| 2 | #9 | 4.2 | 749 | 1 | 4.2 | 1219 | 1 | 0.0877 |  | 95.92 | 5.39 | 85 | MLE  RBF |
|  | #10 | 3.6 | 749 | 1 | 4.2 | 1219 | 1 | 0.0872 |  | 96.27 | 5.02 | 81 | MLE  RBF |
|  | #11 | 3.6 | 749 | 1 | 4.2 | 1194 | 1 | 0.0848 |  | 95.15 | 5.99 | 85 | MLE Matern |
|  | #12 | 4.2 | 762 | 1 | 4.2 | 1257 | 1 | 0.3528 |  | 96.81 | 1.84 | 92 | MAP  RBF |
|  | #13 | 3.6 | 749 | 1 | 4.2 | 1245 | 1 | 0.3351 |  | 85.06 | 1.95 | 83 | MAP  RBF |
|  | #14 | 3.9 | 749 | 1 | 4.2 | 1245 | 1 | 0.2226 |  | 86.13 | 3.53 | 86 | MAP Matern |
| 3 | #15 | 4.2 | 762 | 1 | 4.2 | 1219 | 1 | 0.0737 |  | 96.27 | 4.49 | 96 | MLE  RBF |
|  | #16 | 3.9 | 762 | 1 | 4.2 | 1219 | 2 | 0.0728 |  | 97.36 | 3.31 | 74 | MLE  RBF |
|  | #17 | 4.5 | 787 | 4 | 3.6 | 1270 | 3 | 0.0894 |  | 86.40 | 12.97 | 87 | MLE Matern |
|  | #18 | 3.3 | 762 | 1 | 4.2 | 1181 | 1 | 0.2205 |  | 85.87 | 1.13 | 83 | MAP  PBF |
|  | #19 | 3.3 | 775 | 1 | 4.2 | 1194 | 1 | 0.173 |  | 81.40 | 3.09 | 87 | MAP Matern |
|  | #20 | 4.5 | 800 | 1 | 4.2 | 1270 | 1 | 0.1031 |  | 95.84 | 2.94 | 95 | MAP Matern |
| 4 | #21 | 4.5 | 787 | 2 | 3.9 | 1270 | 1 | 0.0815 |  | 96.70 | 4.37 | 77 | MLE RBF |
|  | #22 | 3.9 | 787 | 3 | 3.9 | 1270 | 1 | 0.1367  0.1153 |  | 109.91  90.92 | 2.21  3.59 | 82 | MAP RBF  MAP Matern |
|  | #23 | 4.5 | 787 | 3 | 3.6 | 1270 | 1 | 0.1366 |  | 92.54 | 2.11 | 75 | MAP RBF |
|  | #24 | 4.5 | 787 | 1 | 4.2 | 1270 | 1 |  | 0.3849  0.2885  0.4672 | 97.03  95.10  85.27 | 3.97  4.70  1.50 | 78 | MLE RBF  MLE Matern  MAP Matern |
|  | #25 | 4.2 | 787 | 1 | 4.2 | 1270 | 1 |  | 0.3839  0.2869  0.4253  0.453 | 97.07  95.10  91.61  103.71 | 3.72  4.70  1.49  0.47 | 79 | All model |
|  | #26 | 3.9 | 787 | 1 | 4.2 | 1270 | 1 |  | 0.3348  0.3898 | 96.39  95.09  91.58 | 4.28  4.70  2.47 | 72 | MLE RBF  MLE Matern  MAP Matern |

**Table S3.** Theoretical and empirical infeasible-proposal rates for unscreened optimization strategies. The table summarizes the theoretical infeasible fraction, empirically observed violation rates over five independent runs, and real fabrication outcomes for each strategy.

| Strategy | Theoretical infeasible fraction | Empirically observed infeasible-proposal rate (5 runs) | Real fabrication outcome |
| --- | --- | --- | --- |
| Random Search | 5.7% | 7.5% | — |
| Space-filling LHS | 5.7% | 5.0% | — |
| Pure BO (unconstrained) | — | 10.0% | iter. 1: 3 candidates non-functional (breakdown) |

The theoretical infeasible fraction (5.7%) represents the analytical probability of sampling physically unstable combinations under independent uniform sampling, calculated based on a 0.029 combined probability of hitting a failure intersection in a single scan pass. The empirically observed rate denotes the actual fraction of violating candidates proposed across five independent runs.

**Figure S1.** (a–d) Evolution of the MLE-Matern model from the 1st to the 4th iteration.

Leave-one-out cross-validation (LOOCV) was used to evaluate model performance, where each of the 26 data points is iteratively left out as a validation sample while the remaining 25 points are used for training. For each model, the prediction errors over all 26 validation cases were summarized using four standard regression metrics: root mean squared error (RMSE), mean absolute error (MAE), weighted absolute percentage error (WAPE), and mean bias error (MBE) are employed to calculate the error. The respective formulas are as follows:

$$\begin{aligned} RMSE=\sqrt{\frac{1}{N}\sum_{n=1}^{N} \left( y_{n}-\hat{y}_{n} \right)^{2}}\#(1) \end{aligned}$$

$$\begin{aligned} MAE=\frac{1}{N}\sum_{n=1}^{N} \left| y_{n}-\hat{y}_{n} \right|\#\left( 2 \right) \end{aligned}$$

$$\begin{aligned} WAPE=\frac{\sum_{n=1}^{N} \left| y_{n}-\hat{y}_{n} \right|}{\sum_{n=1}^{N} \left| y_{n} \right|}\times100\%\#\left( 3 \right) \end{aligned}$$

$$\begin{aligned} MBE=\frac{1}{N}\sum_{n=1}^{N} (y_{n}-\hat{y}_{n})\#\left( 4 \right) \end{aligned}$$

Here, $N$ represents the total number of datasets, while $\hat{y}_{n}$ and $y_{n}$, denote the predicted value of the model and the true value from the experiment, respectively.

**Figure S2.** Calculated accuracies of the four GPR models, SVR model, and KRR model with RMSE, MAE, SMAPE, and MBE metrics. Each metric was estimated by LOOCV.

**Figure S3.** XPS Si 2p spectra of (a) P1, (b) P2, (c) P3, (d) the EAAL-optimized anode (i.e., S7), and (e) P4.

**Figure S4.** Equivalent circuit used for EIS fitting.

**Figure S5.** (a) O/Si ratios derived from the XPS Si 2p spectra, and (b) summarized atomic fractions of the Si oxidation states of the EAAL-optimized anode (i.e., S7), P5, and P6.

**Figure S6.** XPS Si 2p spectra of (a) P5, (b) The EAAL-optimized anode, and (c) P6.

**Figure S7.** (a) Nyquist plots obtained from half-cells assembled, and (b) values in total interfacial resistance (Rtot = RSEI + Rct) obtained from the Nyquist plots for the EAAL-optimized anode (denoted as EAAL in the figure) and P5~P6.

**Figure S8.** Cycling performances of the EAAL- and ETAE-optimized anodes at a current density of 0.5 A g^-1^ after 35 cycles of rate retention test seen in Figure S13.

**Figure S9.** Summarized atomic fractions of the Si oxidation states of the EAAL- and ETAE-optimized anodes.

**Figure S10.** XPS Si 2p spectrum of the ETAE-optimized anode.

**Figure S11.** HRTEM images of (a) as-synthesized graphene-wrapped SiO_2_ nanoparticle before laser annealing and (b) mesorporous SiO_x_ nanoparticle after laser annealing, residing inside the anode (percolated nanoparticles in a vicinity of mesorporous SiO_x_ nanoparticle are electrically conductive carbon blacks incorporated inside the anode)

**Figure S12.** Nyquist plot obtained from the half-cells assembled with the ETAE-optimized anode.

**Figure S13.** Rate performances of the EAAL- and ETAE-optimized anodes.

**Figure S14.** XPS C 1s spectrum of the ETAE-optimized anode.
